# Investigating the mechanism of oridonin against triple-negative breast cancer based on network pharmacology and molecular docking

**DOI:** 10.1371/journal.pone.0332697

**Published:** 2025-12-01

**Authors:** Yuan Zhao, Song Lan, Xinyu Li, Yimin Deng, Limei Sun, Diping Yu

**Affiliations:** 1 The Thyroid Gland and Breast Surgery, Pu’er People’s Hospital, Puer, Yunnan, People's Republic China; 2 Department of Pathology, Pu’er People’s Hospital, Puer, Yunnan, People's Republic China; 3 Department of Respiratory and Critical Care Medicine, Pu’er People’s Hospital, Puer, Yunnan, People's Republic China; Fujian Provincial Hospital, CHINA

## Abstract

Oridonin, a tetracyclic diterpenoid from *Rabdosia rubescens (Hemsl.) Hara,* exhibits various pharmacological actions, such as anti-tumor, anti-infective, and anti-inflammatory properties. However, the underlying pharmacological effects of oridonin on triple-negative breast cancer (TNBC) are yet to be elucidated. This study aims to examine the molecular mechanism and pharmacological impact of oridonin on TNBC using a network pharmacological strategy. Initially, the pharmacological databases and analysis platforms were employed to identify the potential targets of oridonin using the GeneCards website. The process of standardizing gene names involved the conversion of all target genes using the UniProt database. The acquired data was subjected to identify prevalent target genes. Then, these genes were examined using the STRING website to create a protein-protein interaction (PPI) network. In addition to Gene ontology (GO) functional annotation and Kyoto Encyclopedia of Genes and Genomes (KEGG) pathway analysis, a molecular docking analysis was conducted to validate the binding conformation between oridonin and the putative target genes. Among the selected 549 genes, 106 genes were found to interact with TNBC. The KEGG study suggested that the underlying mechanism could potentially be linked to the PI3K/Akt signaling pathway and proteoglycans in cancer. Moreover, molecular docking studies indicated that oridonin exhibited the strongest binding affinity with AKT1 (binding energy: −11.40 kcal/mol) and significant associations with other major targets, including EGFR, NFKB1, MAPK1, and SRC. In summary, the resultant findings based on molecular docking and network pharmacology could demonstrate the potential applicability of oridonin for treating TNBC through pathways like PI3K/Akt signaling.

## 1. Introduction

Triple-negative breast cancer (TNBC) has emerged as one of the most dreadful subtypes of cancer in women, accounting for 15% to 20% of breast cancer cases [1]. Owing to the lack of estrogen, progesterone, and human epidermal growth factor receptors [2], TNBC often responds poorly to chemotherapy, which makes it challenging to treat with endocrine or targeted therapies [1]. TNBC is characterized by its high heterogeneity and aggressiveness [2]. These characteristics contribute to a high recurrence rate and metastatic potential, leading to poor prognosis. Currently, chemotherapy remains the mainstay of treatment; however, the efficacy is often limited due to drug resistance in TNBC cells. Thus, several efforts have been put forward to develop cutting-edge natural treatments that can be utilized to treat TNBC, which is imperative [3].

In recent times, several investigations have demonstrated that Traditional Chinese medicine (TCM)-based herbs and their constituents could exhibit substantial anti-tumor properties. In this context, these mechanisms encompassed impeding the progression of the cell cycle, inducing cellular differentiation, initiating programmed cell death or autophagy, and mitigating the development of resistance to chemotherapy. Along this line, oridonin is a tetracyclic diterpenoid natural compound obtained from the whole herb of the traditional medicinal plant *Rabdosia rubescens (Hemsl.) Hara* belongs to the Labiatae family. The natural compound exhibits various pharmacological actions, such as anti-tumor, anti-infective, and anti-inflammatory properties [4–6]. Previous reports indicated that oridonin exhibited substantial anti-cancer characteristics on diverse cancer cell lines, including lung cancer [7], gastric cancer [8], breast cancer [9], and colorectal cancer [4]. For instance, it was demonstrated that oridonin could hinder the growth and spread of breast cancer by obstructing Notch signaling [9]. In addition, it could reduce the activity of Janus kinase (JAK)/signal transducer of activation (STAT) signaling pathway, restraining epithelial-mesenchymal transition and angiogenesis in thyroid cancer [10]. In another instance, Che et al. [11] showed that treatment of bladder cancer (T24 cells) with different concentrations of oridonin could inhibit transient receptor potential cation channel, subfamily M, member 7 (TRPM7) expression by extracellular signal-regulated kinase (ERK), and AKT signaling pathways, promoting apoptosis and inhibiting the proliferation and migration of bladder cancer. Accordingly, there was a suppression of TRPM7 expression, accompanied by an induction of apoptosis and inhibition of the advancement and spread of bladder cancer. Nevertheless, the precise molecular mechanism of oridonin’s action in treating TNBC remains unclear.

Network pharmacology, a novel approach to drug research, has been formulated by integrating the fields of bioinformatics, systems biology, and pharmacology. This strategy utilizes systems biology and bioinformatics methodologies to comprehensively investigate the network of interactions between disease, target genes, and drugs. Compared to traditional single-target drug research methods, network pharmacology can reveal the synergistic effects of drugs on multiple targets and pathways, thus providing a more comprehensive understanding of their mechanisms. This method is particularly suitable for studying the therapeutic mechanisms of complex diseases such as TNBC [2,12], which involves multiple signaling pathways and biological processes. The network analysis systematically observes the intervention and influence of drugs on the disease network, clarifying the synergistic effect of drugs on various molecular targets within the human body. Further, the acronyms of technical terms are provided with their full explanations upon initial usage. In addition, the field of network pharmacology offers a comprehensive perspective to investigate and elucidate the underlying mechanisms of TCMs. This holistic-adopted approach facilitates the advancement of research and the broadening of clinical applications. Moreover, the molecular docking process uses computational techniques to mimic the interaction between small and bigger molecules, utilizing silico structure theory. Notably, this simulation approach aims to provide valuable insights about the binding site and the complimentary nature of the involved molecules. Owing to the dynamic nature of the field, it utilizes a valuable instrument in structure-based drug design, lead optimization, biochemical pathways, and drug design. Currently, molecular docking methodologies are commonly used in pharmaceutical research to discover novel compounds with therapeutic potential, forecasting the interactions between ligands and targets at the molecular scale and elucidating their conformational relationships.

**Fig 1:**
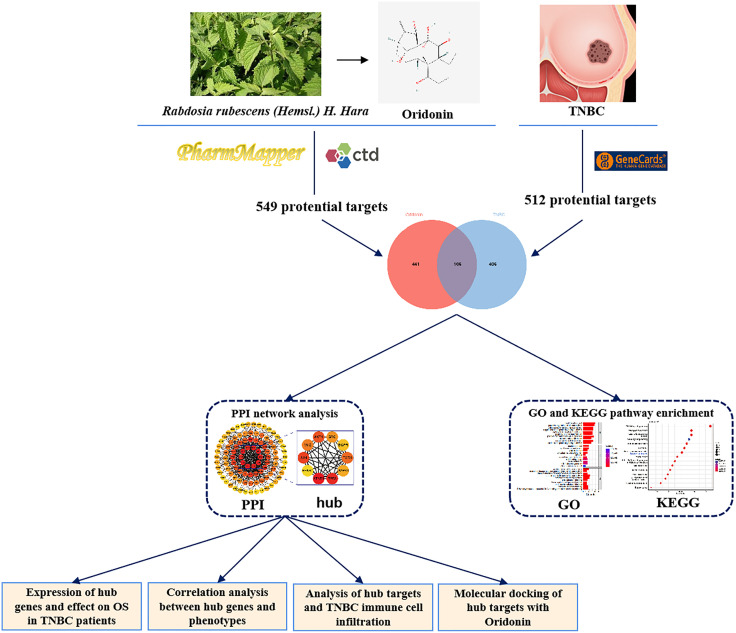
A schematic illustrates the research workflow diagram.

Motivated by these considerations, this study aims to examine the potential pharmacodynamic agents and targets involved in oridonin’s therapeutic effects on TNBC by employing network pharmacology analysis and molecular docking techniques. Moreover, we established a theoretical framework for developing novel pharmaceutical interventions targeting TNBC (**Fig 1**). Initially, the pharmacological databases and analysis platforms were employed to identify the potential targets of oridonin using the GeneCards website. The process of standardizing gene names involved the conversion of all target genes using the UniProt database. The acquired data was subjected to identify prevalent target genes.

## 2. Experimental section

### 2.1. Drug and disease target acquisition

To determine the targets of action of oridonin, the structure-related files of oridonin in different formats were initially obtained by PubChem through TargetNet [13] (http://targetnet.scbdd.com/), Pubchem (https://pubchem.ncbi.nlm.nih.gov), and CTD [14] (https://ctdbase.org/) databases. Further, the oridonin target dataset was generated by standardizing target names and merging duplicate entries using the UniProt database. A dataset of genes related to TNBC was developed by retrieving data from the GeneCards database (https://www.genecards.org./) using the search term “Triple-negative breast cancer”. Finally, the targets of oridonin and TNBC were integrated using a Venn diagram to find possible applications of oridonin for treating TNBC.

### 2.2. Gene function and pathway enrichment analysis

Typically, the Gene Ontology (GO) [15] framework is widely employed to examine the functional enrichment of genes and proteins. Contrarily, the Kyoto Encyclopedia of Genes and Genomes (KEGG) [16] pathway enrichment analysis offers a more comprehensive elucidation of diverse gene and protein activities over GO. The GO and KEGG enrichment analyses of target genes were performed using the R programming language packages “clusterProfiler,” “org.Hs.eg.db,” “enrichplot,” and “ggplot2”. Accordingly, the top 10 biological processes (BPs), cellular components (CCs), and molecular functions (MFs) in GO analysis and the top 15 pathways in the KEGG analysis were screened at *P* < 0.05. The GO and KEGG analyses were plotted using the R platform (https://www.bioinformatics.com.cn).

### 2.3. Construction of PPI and hub targets networks

The online network analysis platform STRING 11.5 [17] (http://string-db.org/) was employed to analyze the network topology. Initially, the standard targets were subjected to the STRING 11.5 database to generate data, with the species “*Homo sapiens*” being chosen for analysis. Further, the Cytoscape version 3.9.0 software was applied to display and analyze the network. Subsequently, the Analyze Network and CytaHubba tools were used to evaluate the hub targets inside the PPI network with degree as the screening criterion.

### 2.4. Correlation analysis between genes and phenotypes

Notably, the VarElect module in GeneCards was used to analyze the correlation between key genes and diseases.

### 2.5. Molecular docking

Typically, molecular docking analysis predicts the binding abilities between small molecular compounds and target genes and their correlation toward drug development. Initially, the two-dimensional (2D) molecular structure of oridonin was obtained from the PubChem database. Further, the Chem3D software was applied to transform the 2D structure into a three-dimensional (3D) structure. Then, hub targets’ crystal structures (n = 10) were obtained from the Protein Data Bank (PDB) library. Prior to performing a molecular docking simulation, the AutoDockTools 1.5.6 program was performed to insert hydrogens and charges, and eliminate all ligands from the protein receptors. The prepared protein receptors and ligands were saved in PDBQT format. The grid size for docking was set to 60 × 60 × 60 points with a spacing of 0.375 Å, centering on the binding site of the target proteins. Molecular docking simulations were carried out using the AutoDock Vina program, applying a Lamarckian genetic algorithm. The resulting binding conformations with the lowest binding energies were analyzed with PyMOL 2.4.1 software to determine the binding interactions. It should be noted that the stronger the binding affinity of oridonin with proteins, the higher the absolute affinity value.

### 2.6. Molecular dynamics simulations

In this study, molecular dynamics (MD) simulations was we employed to explore the dynamic interactions between proteins and ligands in depth. GROMACS software (version 2021.3) was utilized in combination with the CHARMM36 force field for the simulations. The docking files were generated based on the results from molecular docking. During the simulations, appropriate parameters were set, and a 10-nanosecond MD simulation was conducted. Data was recorded every picosecond for subsequent analysis. To evaluate the stability of each complex, we used qtgrace software to visualize root-mean-square deviation (RMSD) and root-mean-square fluctuation (RMSF) results.

### 2.7. Gene expression and survival analysis

The BC gene expression datasets were obtained from The Cancer Genome Atlas Program (TCGA) database, consisting of cancerous and corresponding paracancerous tissue samples, along with patient-related clinical data. Notably, the subjects for analysis were selected by evaluating their clinical information to identify TNBC cases characterized by negative progesterone receptor (PR), negative estrogen receptor (ER), and negative human epidermal growth factor receptor 2 (HER2) conditions. To analyze expression matrices of both normal and tumor groups, the R software was used to extract expression data of potential target genes of oridonin acting on TNBC based on the TCGA database. Further, the edge R and DEGseq packages were employed to analyze and screen DEGs in TNBC and normal tissue samples. After plotting histograms with loglFCl > 1 and *P *< 0.05 thresholds, a survival analysis was performed on possible target genes of oridonin in TNBC using the Kaplan-Meier (KM) Plotter web platform. Eventually, the best nodes were selected while setting negative ER, PR, and HER2 statuses. It should be noted that the genes with *P *< 0.05 in the results could influence TNBC patient survival.

### 2.8. Immune cell infiltration

The CIBERSORT algorithm was employed to analyze the RNA-seq data from individuals diagnosed with TNBC. Initially, the methodology was applied to infer the relative proportions of immune infiltrating cells (n = 22). Further, Spearman’s correlation analysis was conducted between gene expression and immune cell focus.

### 2.9. Statistical analysis

The statistical analysis was conducted using the R program. The data were statistically analyzed using a two-tailed technique, considering a defined significance level of *P* < 0.05.

## 3. Results

### 3.1. Pharmacological screening of anti-TNBC target genes

**Fig 2:**
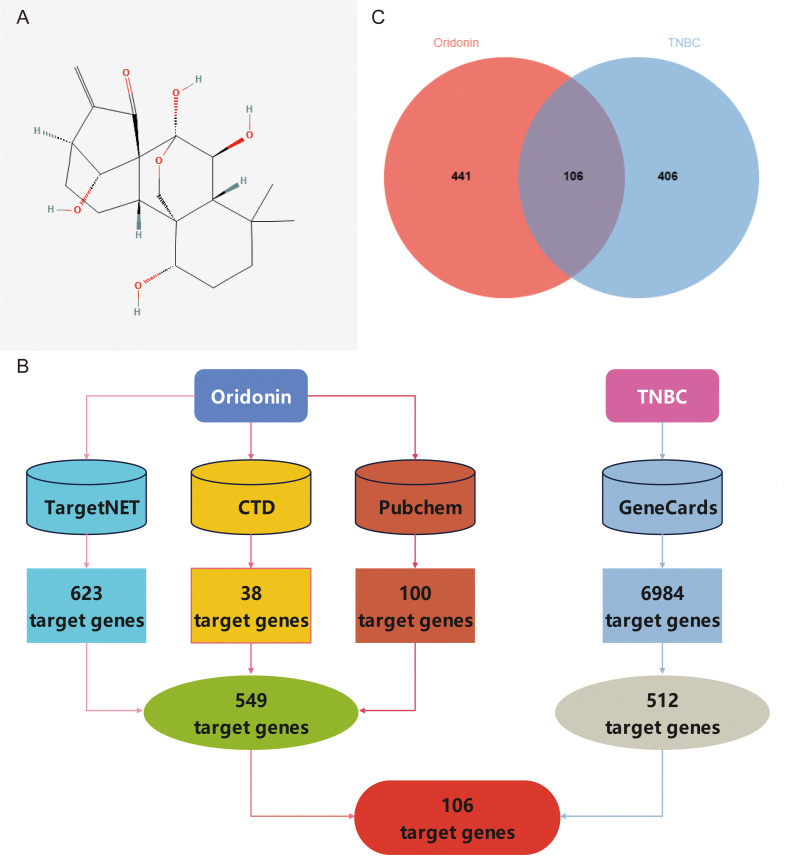
Screening of target genes for anti-TNBC of Oridonin. (A) The molecular diagram shows the 2D structure of the oridonin molecule. (B) The flowchart shows the screened anti-TNBC target genes of oridonin via network pharmacological analysis. (C) The image shows the Venn diagram of target genes of oridonin anti-TNBC obtained by network pharmacological analysis.

Typically, the genetic factors in treating a specific disease play crucial roles in contemporary research in small molecule drug discovery and development. To examine the genes associated with the anti-TNBC effects of oridonin, the target genes of oridonin against TNBC were analyzed using a web-based pharmacology approach. Initially, the two-dimensional structure of Oridonin was acquired from the PubChem database (**Fig 2A**). Subsequently, the TargetNet, CTD, and Pubchem databases were applied to ascertain a total of 623, 38, and 100 genes, respectively, associated with oridonin. In this study, information about a total of 6984 genes about TNBC was initially obtained from the GeneCards database. Further, a subset of genes with a “Relevance score” over 40 was identified, resulting in the selection of 512 genes. Finally, the data of 549 oridonin-related target genes were pooled from TargetNet, CTD, and Pubchem databases, respectively. Moreover, 512 TNBC-associated target genes from the GeneCards database and the intersections of these two databases were considered to finally identify 106 genes shared by oridonin and TNBC (**Fig 2B** and **C**).

### 3.2. Functional and pathway enrichment analyses of common targets

**Fig 3:**
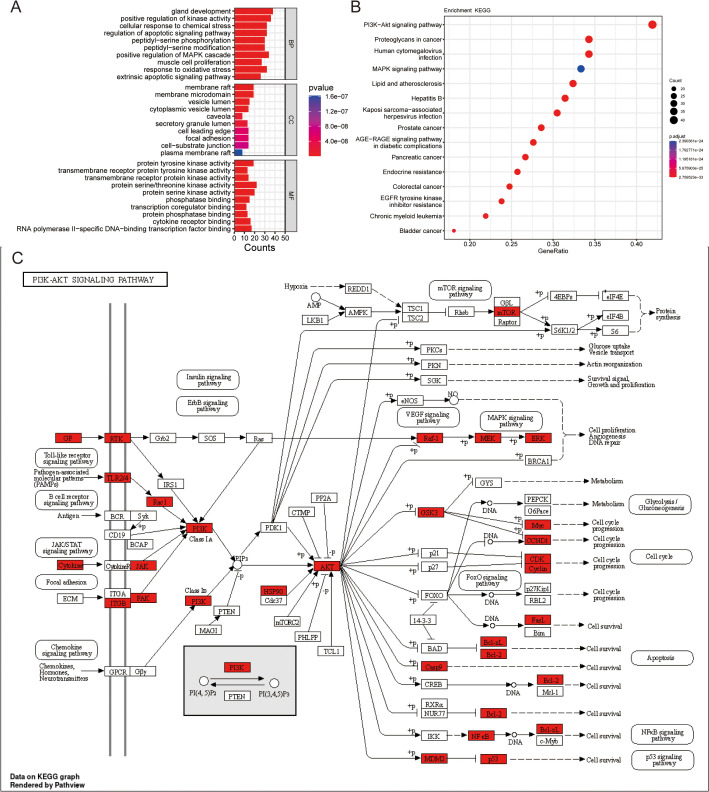
GO and KEGG pathway enrichment analyses. (A) The top 10 enriched Gene Ontology (GO) terms related to biological processes, cellular components, and molecular functions of oridonin’s anti-TNBC target genes. The x-axis indicates the number of genes included in each term, and the y-axis lists the GO terms. The color gradient represents the significance level (p-value) of the enrichment analysis. (B) The top 15 Kyoto Encyclopedia of Genes and Genomes (KEGG) pathways enriched for oridonin’s anti-TNBC target genes. The x-axis indicates the GeneRatio, representing the proportion of genes in the pathway that are target genes of oridonin. The y-axis lists the KEGG pathways. The size of the dots correlates with the number of genes, and the color gradient represents the p-value significance. (C) The target genes involved in the KEGG PI3K/Akt signaling pathway. Red boxes indicate genes targeted by oridonin. The pathway diagram illustrates the interactions and signaling transductions involving these genes.

The KEGG enrichment analysis was performed to assess GO for 106 common targets. Then, it was observed that 4072 results were connected to BPs, in which 2624 were substantially different (*P* < 0.05). Moreover, 260 results were associated with CCs, of which 105 of them were considerably different (*P *< 0.05). Related to MFs, 154 results among 419 were significantly different (*P *< 0.05). The GO results are shown in **Fig 3A** (TOP 10). Concerning BP, these genes exhibited enrichment in processes, including gland development, positive control of kinase activity, cellular response to chemical stress, and regulation of apoptotic signaling pathway peptidyl-serine phosphorylation, among others. The modifications in CCs could mainly affect several cellular components, such as membrane rafts, membrane microdomains, vesicle lumens, cytoplasmic vesicle lumens, and caveolae, among others. The molecular functions encompassed within this category consisted of protein tyrosine kinase activity, transmembrane receptor protein tyrosine kinase activity, transmembrane receptor protein kinase activity, protein serine/threonine kinase activity, and protein serine kinase activity. Moreover, the KEGG results showed that these potential target genes were enriched in a total of 200 pathways. Among these, 172 pathways were significantly different (*P *< 0.05), including PI3K-Akt signaling pathway, proteoglycans in cancer, human cytomegalovirus infection, mitogen-activated protein kinase (MAPK) signaling pathway, as well as lipid and atherosclerosis, among others (**Fig 3B**). The KEGG pathway exhibited the highest enrichment of common target genes, the phosphatidylinositol 3’-kinase (PI3K)-Akt signaling pathway. Based on the KEGG-based transcriptome sequencing results, it could be deduced that the PI3K/AKT signaling pathway could potentially play a substantial role in the progression of anti-TNBC using oridonin. Typically, the PI3K/AKT signaling system is essential in mediating responses to extracellular signals and governing several biological processes, such as cell proliferation, apoptosis, and migration. These findings implied that SAL might exert an anti-EC effect by modulating the PI3K/AKT signaling pathway (**Fig 3C**).

### 3.3. PPI network analysis of common targets

**Fig 4:**
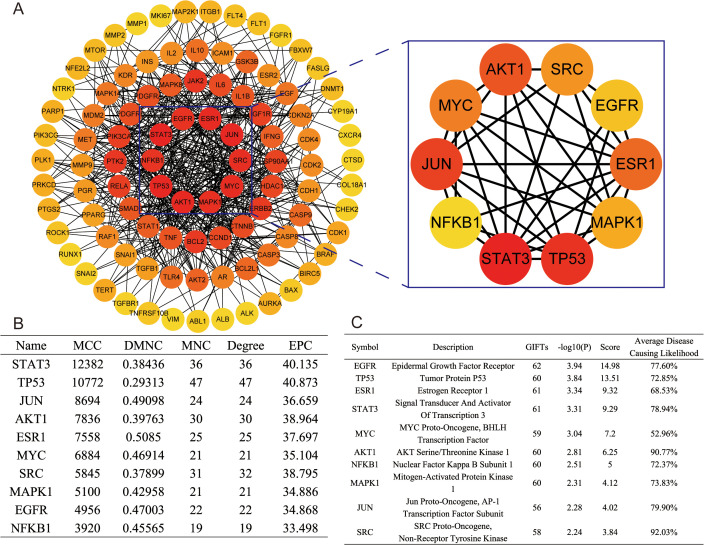
Bioinformatic analysis of PPI-enriched. (A) The draw shows the PPI network of potential Hub target genes. (B) The table presents the information on hub targets in PPI enrichment. (C) The table presents the correlation of the core genes with the TNBC phenotype.

The identification of drug targets is of paramount importance for the comprehensive advancement of pharmaceutical agents. Nevertheless, the specific target of its action in TNBC yet remains to be documented in the literature. In the current study, investigations indicated that oridonin might play a significant regulatory function in the evolution of anti-TNBC. Hence, the STRING database was utilized to investigate the interactions among them (n = 106), in which the often-identified targets were derived from the transcriptomics analysis. Through the application of network pharmacology, these interactions were examined and specifically focused on those with a confidence score exceeding 0.9. Using the software Cytoscape, a comprehensive interaction network was developed. The resultant comprehensive interaction network consisted of 103 nodes and 539 edges, in which the degree value of each node was represented by a darker color, indicating a higher degree. Moreover, the MCC method was employed in the PPI network to identify the top 10 hub targets, including STAT3, TP53, JUN, AKT1, ESR1, MYC, SRC, MAPK1, EGFR, and NFKB1 (**Fig 4A** and B). Further, the gene-phenotype correlation analyses were performed to assess the correlation between key target genes and TNBC. The scores of the 10 key targets were evaluated using the VarElect module in GeneCards. It was observed from the assessment results that a clear positive connection was revealed between all 10 central targets and TNBC. As depicted in **Fig 4C**, the Average Disease Causing Likelihood of SRC, AKT1, and TNBC exceeded 90%. In contrast, the Average Disease Causing Likelihood of EGFR, TP53, STAT3, NFKB1, MAPK1, JUN, and TNBC was above 70%.

### 3.3. Analysis of the hub targets

**Fig 5:**
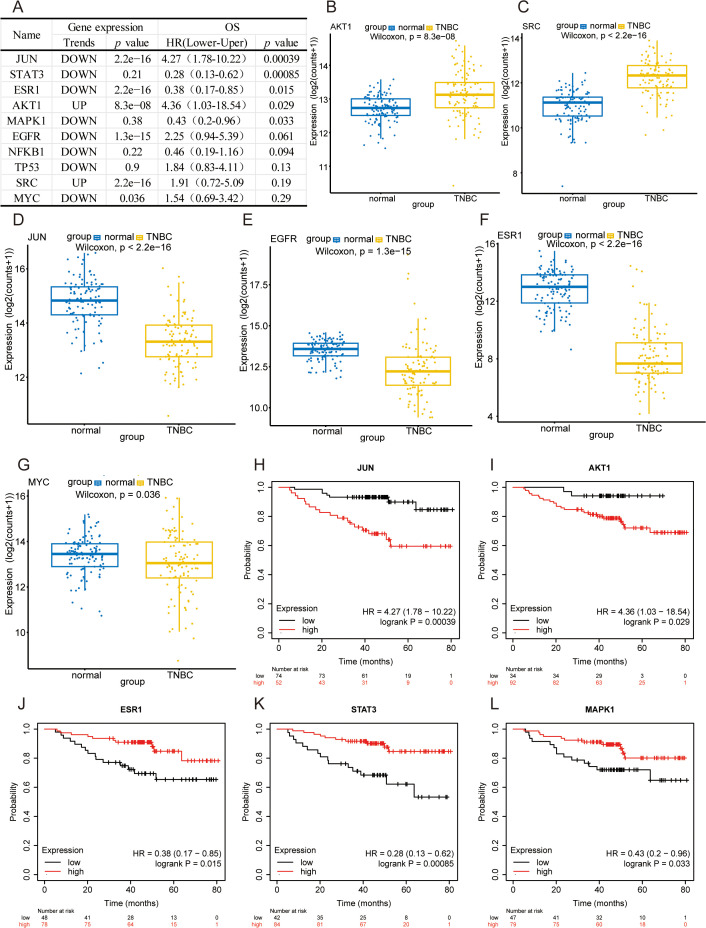
The analysis of hub targets. (A) The table presents the expression of hub targets in TNBC and the correlation analysis with the overall survival time of patients. (B-G) The graphs show the expression maps of AKT1, SRC, JUN, EGFR, ESR1, and MYC in TNBC patients and normal tissues. (H-L) The graphs show the target expression levels in TNBC and the correlation analysis with the patient’s overall survival time, affecting TNBC patients’ survival of JUN, AKT1, ESR1, ATAT3, and MAPK1 expression.

**Fig 6:**
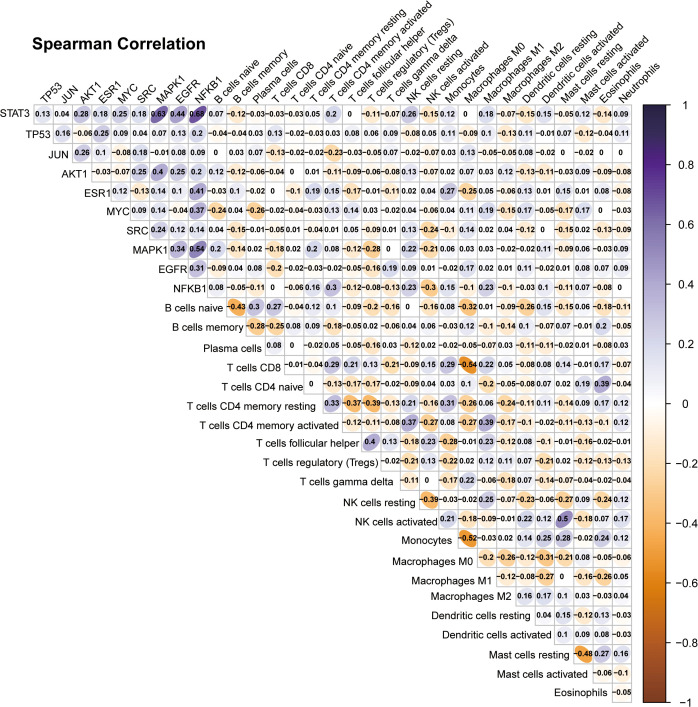
The image shows the oridonin interference with hub targets of TNBC concerning tumor immune cell infiltration.

Further, several investigations were undertaken to examine the expression of hub targets in patients with TNBC and its influence on their survival outcomes (**Fig 5A**). It was observed that the expression levels of AKT1 and SRC were significantly different between patients with TNBC and their corresponding paraneoplastic tissues. In contrast, the expression levels of JUN, EGFR, ESR1, and MYC were considerably lower in the cancerous tissues than in the paraneoplastic tissues of TNBC patients (**Fig 5B**–**G**). In addition, it was observed that high expression levels of several genes, including JUN and AKT1, could be associated with poorer survival rates in TNBC patients. Conversely, the low expression levels, *i.e.,* STAT3, ESR1, and MAPK1, could predict poorer survival in TNBC patients (**Fig 5H**–**L**). Considerably, the immunological molecular pathways associated with oridonin intervention in TNBC were investigated. In addition, the correlation between 10 key target genes and the infiltration of immune cells into the tumor microenvironment was examined. It was observed from the findings that a positive correlation was established between STAT3 and resting natural killer (NK) cells, as well as activated CD4 memory T cells. Conversely, a negative association was observed between JUN and triggered CD4 memory T cells. Typically, the NFKB1 gene could be linked to the activation of CD4 memory T cells, the resting state of NK cells, and the M1 phenotype of macrophages. In addition, there existed a negative correlation between NFKB1 and the activation of NK cells. Moreover, a positive correlation existed between EGFR and Macrophages M0, while a negative correlation existed between EGFR and CD8 T cells. In this study, it was observed that the expression of MAPK1 exhibited a positive correlation with B cells naïve, CD4 memory resting T cells, and resting NK cells and a negative correlation with T cells regulatory (Tregs) and activated NK cells. Nevertheless, a negative correlation was observed between SRC and the activation of NK cells. Moreover, the expression of MYC was positively correlated with Macrophages M1 and was negatively correlated with B cells naïve and plasma cells. The ESR1 expression exhibited a positive correlation with Monocytes M0 and a negative correlation with CD8 T lymphocytes. A positive correlation was observed between ESR1 and monocytes, while a negative correlation was found between ESR1 and macrophage M0 (**Fig 6**).

### 3.4. Molecular docking

**Fig 7:**
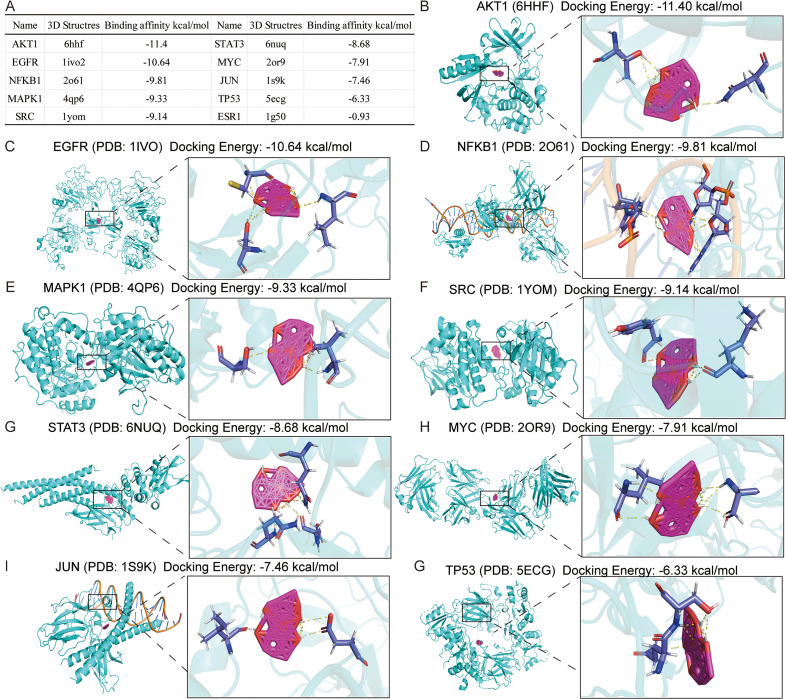
Molecular docking results of hub targets and Oridonin. **(A)** The table shows the molecular docking results of oridonin and hub targets. **(B-G)** The images indicate the molecular docking visualization results of oridonin binding to AKR1, EGFR, NFKB1, MAPK1, SRC, STAT3, MYC, JUN, and TP53.

Finally, molecular docking was employed to clarify the specific target of oridonin against TNBC with hub targets. It should be noted that the binding energy between small molecule ligands and protein receptors could denote their affinity level, in which the lower the energy, the better the affinity and stability. As depicted in **Fig 7A**, oridonin exhibited the most favorable binding energy towards AKT1, with EGFR, NFKB1, MAPK1, SRC, STAT3, MYC, JUN, and TP53 in descending order. The decreased binding energies suggested a strong binding affinity between oridonin and the respective targets. Therefore, these targets and their influence on structural changes might regulate the corresponding signaling pathways. **Fig 7B**–**G** display the docking structures of oridonin with its target proteins, including AKT1, EGFR, NFKB1, MAPK1, SRC, STAT3, MYC, JUN, and TP53.

### 3.5. Molecular dynamics simulations

**Fig 8:**
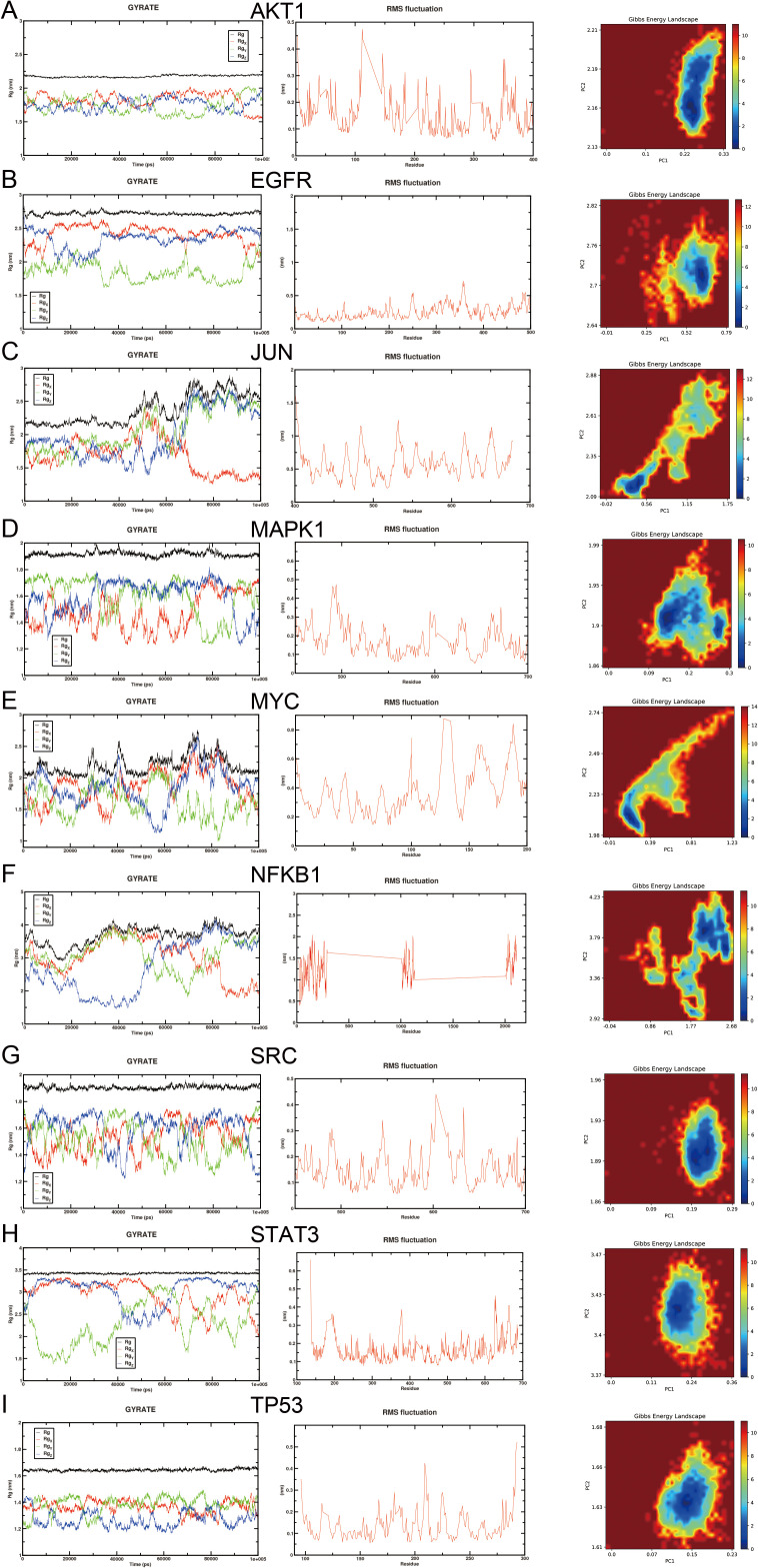
Molecular docking and molecular dynamics simulation results for oridonin with key TNBC targets. (A-I) Gyration and RMSF plots, Gibbs energy landscape.

To further validate the binding interactions and stability of these complexes, molecular dynamics (MD) simulations were conducted. Fig 8A–I display the protein gyration and root mean square fluctuation (RMSF) plots along with the Gibbs energy landscapes for complexes of oridonin with its target proteins including AKT1, EGFR, NFKB1, MAPK1, SRC, STAT3, MYC, JUN, and TP53. The MD simulations reveal that oridonin maintains stable interactions with these targets over time, indicating its potential as a robust inhibitory agent. Specifically, the AKT1-oridonin complex showed the least fluctuation and most stable conformation, corresponding to the lowest binding energy identified in the docking studies. The Gibbs energy landscapes further illustrate the conformational stability of these complexes, with energy minima indicating favorable binding conformations.

## 4. Discussion

TNBC, with a complicated and varied nature, is often characterized by the absence of ER, PR, and HER2 expressions. The TNBC condition exhibits early onset, significant invasiveness, unfavorable prognosis, premature local recurrence, and distant metastases, accounting for increased mortality rates and a decreased median survival rate compared to non-triple TNBC. The most prevalent treatment modality for this type of cancer includes a combination of surgery, chemotherapy, and radiotherapy. Due to low survival rates, there is an urgent need to develop effective targeted therapy options. Notably, natural products offer more advantages compared to small-molecular drugs-based conventional chemotherapy. Accordingly, these agents can engage with numerous targets, exhibiting significant therapeutic efficacy while demonstrating minimal systemic toxicity. Along this line, the emergence of novel moieties has garnered considerable attention from researchers toward developing anti-cancer medicines.

Previous studies on oridonin’s anti-triple-negative breast cancer (TNBC) effects have primarily focused on specific molecular mechanisms or structural modifications. For example, oridonin has been shown to induce apoptosis through DR5/p21 upregulation [18,19], inhibit metastasis by suppressing MMP-2/9 [20], or achieve immunoregulation by blocking Treg differentiation [21]. In contrast, our study employs a systematic network pharmacology approach to uncover the multi-target regulatory network of oridonin. The field of network pharmacology holds promise in elucidating the mechanisms of drug action. Along this line, integrating molecular docking techniques can assist in identifying innovative therapeutic agents that can specifically target disease-associated genes. In this study, the network pharmacology approach was employed to identify target genes for treating TNBC with oridonin. Initially, a PPI network consisting of 106 overlapping genes was subsequently developed. Further, we analyzed KEGG pathways to investigate the interplay among target genes implicated in oridonin therapy against cancer. The KEGG results revealed that oridonin could target to treat TNBC, encompassing PI3K/Akt signaling, proteoglycans in cancer, human cytomegalovirus infection, MAPK signaling, lipid metabolism, and atherosclerosis, among others. Accordingly, the PI3K/Akt signaling system is the most gene-enriched pathway among the several signaling pathways. This crucial cellular signaling pathway could facilitate metabolic processes, cell proliferation, and cell survival, affecting the viability of cells. It should be noted that the classical pathway could be commonly linked to various malignancies [22]. In a case, it was demonstrated that the dysregulation of PI3K/Akt signaling could also be a significant feature of TNBC [23]. Inhibiting the activity of this pathway could lead to anti-tumor efficacy [24]. These studies implied that the PI3K/AKT signaling pathway played a pivotal role in the development of TNBC. Consequently, the inhibition or blockade of this system might serve as a promising molecular target for the advancement of novel therapeutic agents.

To investigate the molecular mechanism and possible targets of oridonin in inhibiting the growth of TNBC, a PPI network analysis was established based on a set of 106 genes. The PPI network results revealed probable key target genes of oridonin in the treatment of TNBC, such as STAT3, TP53, JUN, AKT1, ESR1, MYC, SRC, MAPK1, EGFR, and NFKB1. Notably, several genes associated with these notified proteins (n = 10) have previously been associated with the initiation and advancement of TNBC. Along this line, previous studies established the significance of the SRC pathway in driving TNBC [25]. Moreover, the signaling mechanisms implicated in SRC could potentially impact the proliferation, survival, migration, and invasion of cancer cells [26]. Moreover, the expression of MAPK1 could be significantly increased in TNBC [27]. In another case, it was demonstrated that AKT1, also known as protein kinase B, played a pivotal role in tumor formation in TNBC [28]. The STAT3 protein was identified as a therapeutic target in TNBC due to its substantial involvement in tumor growth, metastasis, and developing breast cancer stem cell-like properties [29]. NFKB1 belongs to the (NF)-κB gene family and represses inflammation, aging, and cancer [30]. In TNBC, the expression levels of EGFR were higher than in non-TNBC (OR = 6.88, *P *< 0.00001), making it a potential biomarker for targeted therapy and prognosis in TNBC [31]. EGFR inhibition prevents cancer stem cell clustering and lung metastasis in TNBC [32]. In this regard, the MAPK1 gene, which exhibits high expression levels in TNBC, plays a significant role in cancer development, which can be linked to an unfavorable prognosis [27]. In a case, it was demonstrated that inhibiting the phosphorylation of MAPK1 effectively reduced tumor growth in TNBC [33]. The inhibition of interferon signaling by MYC was observed to contribute to immunosuppression in TNBC [34,35]. In addition, this phenomenon could be associated with the development of resistance to immune checkpoint inhibitors [36]. Furthermore, it was observed that patients with TNBC showed a notable rise in the occurrence of ESR1 mutations, which could be positively correlated with TNBC [37].

In this study, TCGA data analysis demonstrated that AKT1 and SRC expression levels were significantly higher in TNBC patients compared to paraneoplastic tissues. Moreover, JUN, EGFR, ESR1, and MYC expression levels in TNBC patients were considerably lower than in paraneoplastic tissues. Furthermore, it was observed that high expression levels of numerous genes, including JUN and AKT1, were related to lower survival rates in TNBC patients. Contrarily, decreased STAT3, ESR1, and MAPK1 expression levels could indicate reduced survival rates among patients with TNBC. In addition, a series of molecular docking experiments were performed, focusing on 10 key target genes. The findings revealed that oridonin could bind effectively to all 10 target genes, with the lowest binding energy noted for AKT1, followed by EGFR, NFKB1, MAPK1, SRC, STAT3, MYC, JUN, and TP53. These findings suggested that oridonin might exert anti-tumor effects by inhibiting key targets and their related signaling pathways.

## 5. Conclusion

In summary, this study has provided an initial examination of oridonin’s anti-TNBC mechanism using a network pharmacology approach. The analysis identified 106 genes potentially interacting with TNBC, with key pathways such as the PI3K/Akt signaling pathway and proteoglycans in cancer being highlighted as significant. Molecular docking studies revealed that oridonin exhibited strong binding affinity with key proteins involved in TNBC, including AKT1 (binding energy: −11.40 kcal/mol), EGFR, NFKB1, MAPK1, and SRC. These findings suggest that oridonin can modulate critical signaling pathways and protein interactions in TNBC, underscoring its potential as a therapeutic agent. The theoretical foundation laid by this study paves the way for future experimental validations, which are essential to confirm these interactions and mechanisms in vitro and in vivo. Despite the limitations of database-dependent analysis and the need for experimental verification, our results indicate that oridonin holds promise for the development of new treatments for TNBC.

## 6. Limitations

Despite the valuable insights gained from our study, several limitations must be acknowledged. Our findings heavily rely on publicly available databases such as GeneCards and STRING. The variability and quality of the integrated data in these databases may introduce potential biases. Additionally, the dynamic nature of these databases, which are periodically updated, could affect the reproducibility of our results. Our study primarily utilizes computational methods such as network pharmacology and molecular docking, lacking direct experimental validation. While these techniques are powerful for hypothesis generation, the predicted interactions and proposed mechanisms require further validation through in vitro and in vivo experiments to confirm their biological relevance. Network pharmacology and molecular docking simplify complex biological systems into computational models, potentially overlooking intricate regulatory networks and other cellular processes that influence the pharmacological actions of oridonin.

## Supporting information

S1 File Code. (TXT)
